# Wearable technology-based metrics for predicting operator performance during cardiac catheterisation

**DOI:** 10.1007/s11548-019-01918-0

**Published:** 2019-02-07

**Authors:** Jonathan Currie, Raymond R. Bond, Paul McCullagh, Pauline Black, Dewar D. Finlay, Stephen Gallagher, Peter Kearney, Aaron Peace, Danail Stoyanov, Colin D. Bicknell, Stephen Leslie, Anthony G. Gallagher

**Affiliations:** 1 School of Computing, Jordanstown Campus, Ulster University, Shore Road, Newtownabbey, BT37 0QB Northern Ireland UK; 2 School of Nursing, Magee Campus, Ulster University, Londonderry, BT48 7JL Northern Ireland UK; 3 School of Engineering, Jordanstown Campus, Ulster University, Londonderry, BT48 7JL Northern Ireland UK; 4 School of Psychology, Coleraine Campus, Ulster University, Cromore Road, Coleraine, BT52 1SA Northern Ireland UK; 5 Application of Science to Simulation Based Education and Research on Training (ASSERT) Centre, University College Cork, Cork, Ireland; 6 Clinical Translational Research and Innovation Centre (C-TRIC), Londonderry, Northern Ireland UK; 7 University College London, London, UK; 8 Imperial College London, London, UK; 9 NHS Highland, NHS Scotland, Edinburgh, UK

**Keywords:** Surgical simulation, Simulation-based training, Eye tracking, Wearable technology, Attentional capacity

## Abstract

**Introduction:**

Unobtrusive metrics that can auto-assess performance during clinical procedures are of value. Three approaches to deriving wearable technology-based metrics are explored: (1) eye tracking, (2) psychophysiological measurements [e.g. electrodermal activity (EDA)] and (3) arm and hand movement via accelerometry. We also measure attentional capacity by tasking the operator with an additional task to track an unrelated object during the procedure.

**Methods:**

Two aspects of performance are measured: (1) using eye gaze and psychophysiology metrics and (2) measuring attentional capacity via an additional unrelated task (to monitor a visual stimulus/playing cards). The aim was to identify metrics that can be used to automatically discriminate between levels of performance or at least between novices and experts. The study was conducted using two groups: (1) novice operators and (2) expert operators. Both groups made two attempts at a coronary angiography procedure using a full-physics virtual reality simulator. Participants wore eye tracking glasses and an E4 wearable wristband. Areas of interest were defined to track visual attention on display screens, including: (1) X-ray, (2) vital signs, (3) instruments and (4) the stimulus screen (for measuring attentional capacity).

**Results:**

Experts provided greater dwell time (63% vs 42%, *p* = 0.03) and fixations (50% vs 34%, *p* = 0.04) on display screens. They also provided greater dwell time (11% vs 5%, *p* = 0.006) and fixations (9% vs 4%, *p* = 0.007) when selecting instruments. The experts’ performance for tracking the unrelated object during the visual stimulus task negatively correlated with total errors (*r* = − 0.95, *p* = 0.0009). Experts also had a higher standard deviation of EDA (2.52 µS vs 0.89 µS, *p* = 0.04).

**Conclusions:**

Eye tracking metrics may help discriminate between a novice and expert operator, by showing that experts maintain greater visual attention on the display screens. In addition, the visual stimulus study shows that an unrelated task can measure attentional capacity.

*Trial registration* This work is registered through clinicaltrials.gov, a service of the U.S. National Health Institute, and is identified by the trial reference: NCT02928796.

## Introduction

Patient safety and the mitigation of medical errors are of growing importance [1]. Poor decision-making and lack of skill in clinical procedures can be significant factors in many of the errors that are reported [2]. Research into clinical skills would suggest a critical role for ‘continual practice’ and maximising training time to reach an ‘appropriate’ level of performance [3]. Simulation-based training has demonstrated that skills can be acquired as well as measured without the need to ‘learn on real patients’ [4, 5]. Many healthcare tasks and procedures can be simulated using computer technology for training purposes and provide novices with a way to improve or maintain their skills [6–8]. In addition to technical skill acquisition, we know that the errors made in the clinical environment are also related to non-technical skills [9] and hence there is a need to understand the relationship between skill and cognitive load during procedures. For example, a high cognitive load may affect the non-technical leadership skills of the operator in the clinical environment.

### Eye tracking in medical research

One interest in measuring performance is investigating the link between visual attention (eye gaze) and clinical performance. This domain investigates whether an operator’s eye gaze behaviour is correlated with their level of competence during a clinical procedure [10–14]. The ‘mind-eye hypothesis’ [15] states that visual attention can indicate cognitive activity [16–18]. Put differently, where someone looks can be indicative of their cognitive experience and thus their level of expertise, situational awareness, uncertainty and perhaps the likelihood that their future actions could cause harm to a patient. A recent study with surgical tasks [11] was shown to discriminate between novices and experts using eye tracking metrics.

### Attentional capacity

Clinical decision-making is comprised of many steps including perception, attention, information processing, information storage (including organisation) and then knowledge retrieval from long-term memory at the appropriate time [19]. One aspect of cognition that has received no consideration in related literature is ‘attention’, yet this is of paramount importance to the interventional cardiologist who is learning a new set of skills. Attention refers to the ability to cognitively focus on an object or activity. It is well known that humans have a limited attentional capacity [20]. The human mind can only attend to a finite amount of information at any given time. When a novice clinical operator is acquiring new skills, they use almost all of their attentional resources to monitor what their hands are doing in addition to the spatial judgments and clinical decision-making. This results in limited ‘additional’ attentional capacity for the novice [21] and hence why this study involves the aforementioned visual stimulus task.

This study aims to (1) use wearable technology to determine metrics that could be used to auto-assess operator and procedural performance and (2) to determine whether a visual stimulus task can be used to measure attentional capacity and whether performance of this task is associative to operator errors. Both objectives were carried out using a state-of-the-art, high-fidelity, full-physics VR simulator which provided the means for recording the procedural performance of interventional cardiologists. This work could lead to ‘smart operating rooms’ that can provide live metrics on individual and team performances, providing critical automated analytical feedback.

Ethical approval for this study was granted across the island of Ireland: (1) Ulster University (ref: FCEEFC 20160630), (2) University College Cork (ref: ECM 4 (g) 09/08/16).

## Methods

This study involved investigating the use of (1) eye tracking, (2) psychophysiological monitoring and (3) attentional capacity in surgical simulation-based assessment (specifically in coronary angiography). We recorded data from two different groups of interventional cardiologists to test the significance of metrics in discriminating between novices and experts. Data collection took place in the ASSERT Centre, University College Cork.

### Study components

The study was comprised of a surgical simulator with simulated patient cases, eye tracking glasses and an E4 wristband for monitoring the operator’s psychophysiology. For the visual stimulus task, an additional LCD display screen was used to display the playing cards.

#### Simulated coronary angiography

A Mentice VIST-Lab^1^ and VIST G5 software (developed by Mentice, Sweden) provided the simulated coronary angiography cases (model details: VIST G5 + VIST-C LD, Coronary PRO v2.3.3, Coronary Angiography v1.3.3 and Coronary Educator v1.1.2). Two cases were assessed by a teaching- and consultant-level interventional cardiologist. One case allowed the participant to practise with the system, and the second case was the primary data collection session. Each participant was allocated ‘up to 30 min’ to practise using the first case allowing the participant to gain a level of familiarity with the simulator. The investigator provided a demonstration on how to use the simulator. Participants were tasked with taking nine views controlling the C-arm:

- *Right Coronary Artery (RCA)*
  - Left Anterior Oblique (LAO) 30°, Cranial 15°
  - Right Anterior Oblique (RAO) 30°
  - Anteriorposterior (AP)
- *Left anterior descending (LAD)*
  - AP
  - RAO 30°, Caudal 30°
  - RAO 10°, Cranial 40°
  - LAO 50°, Cranial 30°
  - LAO 40°, Caudal 30°
  - Lateral

#### Wearable technologies

SMI^2^ eye tracking glasses were used to measure visual attention during procedures. The glasses allow the participant to move freely while performing the procedure; while capturing temporal and spatial metrics. Empatica’s E4^3^ wristband provided real-time measurements of the participant’s heart rate, inter-beat intervals (or heart rate variability), EDA (4 Hz), skin temperature (4 Hz) and an accelerometer (32 Hz).

#### Visual stimulus card task to measure attentional capacity

To measure attentional capacity by proxy, each participant was given an additional visual stimulus to monitor (playing cards) and tasked to verbally respond with the word ‘heart’ when a given playing card (queen of hearts) appeared on the LCD screen. It was made clear that the priority should be performing the procedure but to undertake this additional task if they could. Two variations of the stimulus were provided, one for each of the two attempts. The first acted as a baseline measurement with less additional attention required, and the second performance demanded greater attentional capacity. We increased the number of cards the participant could examine per minute between the first and second performance.

This aspect of the study is based on the works from Weaver [22] and Smith [23]. In Smith’s experiment, a playing card provides 5.7 bits/items of information. Using this measurement, the difference for information output between the stimulus tasks presented during the first and second procedures can be quantified. However, the exposure duration of the playing card is also important and the 2 s exposure duration was determined to be appropriate for this study.

Participants are asked to examine the cards and detect a specific card that they were instructed to verbally acknowledge. Both variations (see ‘Appendix A’ for further detail) have the same design: continual blocks of 20 s with one card that they are instructed to verbally acknowledge. Within these 20 s blocks, ten different cards would appear for 2 s each. Using a random number generator, the random position (within the 20 s block) of the specific card would be continually changed according to an integer 1–10 (referencing its position in the block). This approach semi-randomised the appearance of the playing card while guaranteeing that the participant would have three cards to acknowledge every 60 s. The first performance attempt only provided three playing cards (5.7 bits/item) exposed for 2 s each and therefore an information output of 17.1 bits per 60 s. In contrast, the second performance stimulus card involved a continuous sequence of cards and had information output of 171 bits every 60 s.

### Protocol

The protocol is comprised of four stages: (1) demonstration of the VIST-Lab simulator, (2) setting up the wearable technology, (3) participant attempts the first task and (4) participant attempts the second task. Details are as follows:

#### Explanation and demonstration of the VIST-Lab simulator

- Participants were informed that a 0.035 guide wire and 5F catheter with a contrast syringe were already connected for use.
- C-Arm controls to facilitate different views were demonstrated. They were asked to record nine views.
- They were shown how to start the case and select instruments.
- They were provided with a practice case and given up to 30 min, allowing for familiarity with the simulator.

#### Assistance with wearable technology

- Before the main procedure, it was necessary to calibrate the eye tracking glasses and begin recording data for both wearable devices.
  - Wristband
    - Once comfortably fitted, wristband was switched on, and using an iOS application, the recording session was initialised via Bluetooth.
  - Eye tracking glasses
    - Once comfortable, the glasses were connected via USB to the portable recording device.
    - Three-point calibration was completed.

### Data analysis

#### Procedural performance

The following performance metrics were exported from the VIST simulator after each session:

- Performance duration (minutes)
- Total errors
  - Type 1: vessel wall scraping
  - Type 2: moving without wire
  - Type 3: too deep in ostium
  - Type 4: wire in small branch
- Wire and catheter use (including counts for each time selected and subsequently detected entering the simulator)

#### Stimulus card task

Using laboratory cameras and eye tracking footage, the cards that were correctly acknowledged by each participant in each performance were counted against all stimulus cards that appeared. A percentage of correctly acknowledged cards were used as an assessment metric.

#### Eye tracking metrics

**Fig. 1:**
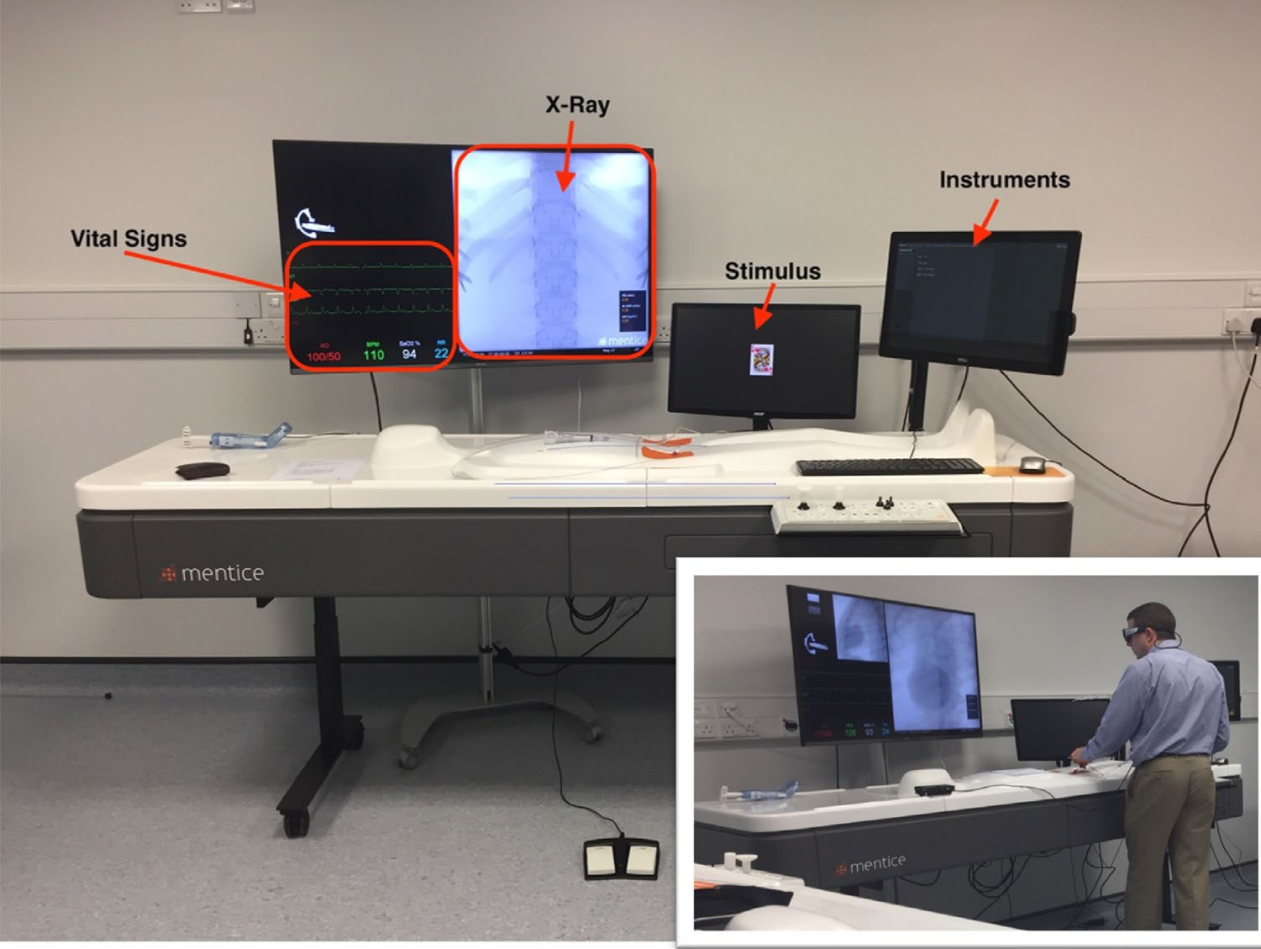
Main image: Mentice VIST-Lab simulator, with the four AOIs identified. Bottom right: a participant during procedural performance, wearing eye tracking glasses connected to the portable recording device placed to the left on the simulator table and wearing the Empatica’s E4 wristband on their wrist (hidden)

Four AOIs were defined as the instruments selection screen, the stimulus screen displaying the cards, the X-ray and the vital signs (see Fig. 1). Eye gaze metrics are derived from fixations and saccades. A fixation is when the participant is fixating on single location using their fovea vision, and a saccade can be a vector between two fixations or rapid movements between fixations [24]. The following eye tracking metrics were calculated which have been used in similar studies [25–28]:

- AOI specific metrics: dwell %, fixation %, first fixation duration (ms).
- General eye tracking (non-AOI): fixation frequency, saccade frequency and saccade latency (ms).
- AOI Fixation Transition Counts.

Fixation transitions count the direct switching of fixations from one AOI to another. Additionally, the counts for transitions between AOIs were totalled into a new metric called total transitions. Another metric was developed using total transitions against procedure duration, i.e. fixation transition frequency (transitions between AOIs per second).

#### Wristband measurements

Measurements recorded from the E4 wristband include heart rate (bpm), inter-beat interval (SD of inter-beat intervals taken as heart rate variability), EDA (micro-Siemens or µS) and skin temperature (°C) and triaxial accelerometry (*X-*, *Y-*, *Z*-axis values at 32 Hz). From the latter, we computed the acceleration magnitude (ACC) using Euclidian distance.

#### Statistical methods

The R programming language was used for the data analytics. Summary statistics for groups are presented as mean and standard deviation (mean ± SD). Different significance tests were chosen to perform depending on (1) data distribution: Mann–Whitney *U* test if non-normal distribution, and (2) unequal/equal variance: Welch *t* test if unequal, Student’s *t* test if equal. All significance tests reported as *p* values were either Mann–Whitney *U* or Welch tests as no equal variances were found. Either the Pearson product moment coefficient (*r*) or the Spearman rank-order correlation coefficient (*ρ*) was used for correlation analysis depending on the normality of the variables. The Shapiro test was used for normality testing in this instance (null hypothesis is that data are normally distributed). Also, Bonferroni-corrected alpha values are presented for transparency.

## Results

Table 1 describes the demographics of the novices and experts in this study.

**Table 1:** Participant demographic information

Demographic	Novice	Expert
Sex	Female = 3 | male = 4	Female = 0 | male = 7
Experience (years)	2.8 ± 1.8	19.9 ± 5.9
Minimum coronary angiograms (annually)	113 ± 91	464 ± 225
“Prior experience with simulation-based training?”	No = 4 | yes = 3	No = 1 | yes = 6
“Prior experience with the study surgical simulator?”	No = 7 | yes = 0	No = 6 | yes = 1
Left-/right-handed	Left = 1 | right = 6	Left = 2 | right = 5
Practice time used	28 ± 4	19 ± 9

Novices had a mean experience in years of 2.8 ± 1.8 versus 19.9 ± 5.9 for experts (*p* < 0.001). Novices had participated in a mean 113 ± 91 coronary angiogram cases in past 12 months versus 464 ± 225 for experts (*p* < 0.01). Experts had more experience in simulation-based training (86% vs 43%). Almost all participants had never used the VIST-Lab simulator (0% vs 14%, 7% in total). Participants were also asked to declare whether they were left- or right-handed (1L and 6R vs 2L and 5R). The only females in the study (n = 3) were novices. Experts were more likely to signal ‘early’ (before 30 min was complete) that they were ready to begin the next case. Experts had a mean practice time of 19 min compared with 28 min for novices (*p* = 0.04).

### Procedural performance

Table 2 presents the key metrics for procedural performance for both attempts. Table 3 presents changes in errors and the stimulus task card acknowledgement %, either improvement or deterioration, between the first and final attempt. It is notable that experts increase their total errors compared with novices, along with a poorer card acknowledgement rate.

**Table 2:** Group comparison: procedure performance metrics

Procedure performance metric		First attempt		*p*	Final attempt		*p*
		Novice	Expert		Novice	Expert	
Performance duration (minutes)		15 ± 5	11 ± 6	0.20	13 ± 5	13 ± 6	0.98
Total errors		11 ± 9	9 ± 6	0.80	11 ± 6	15 ± 7	0.30
Error type 1	Vessel wall scraping	1 ± 3	0 ± 0	0.90	1 ± 2	2 ± 2	0.20
Error type 2	Moving without wire	8 ± 6	7 ± 6	1.00	8 ± 5	12 ± 8	0.30
Error type 3	Too deep in ostium	1 ± 1	1 ± 1	0.60	1 ± 0	1 ± 1	0.80
Error type 4	Wire in small branch	1 ± 1	0 ± 1	0.90	1 ± 1	0 ± 0	0.50
Card acknowledgement %		72 ± 31	76 ± 20	0.70	75 ± 21	74 ± 20	0.90
Wire/catheter count		3 ± 0	3 ± 0	N/A	3 ± 0	3 ± 1	0.70
Wire/catheter re-entry		5 ± 5	3 ± 1	0.30	3 ± 2	4 ± 3	0.40

**Table 3:** Group comparison: key metric changes between attempts

Metric change	Novice	Expert	*p*
Total errors	0 ± 8	+ 6 ± 10	0.20
Card acknowledgement %	+ 4 ± 28	− 2 ± 22	0.70

### Stimulus task and total errors

**Fig. 2:**
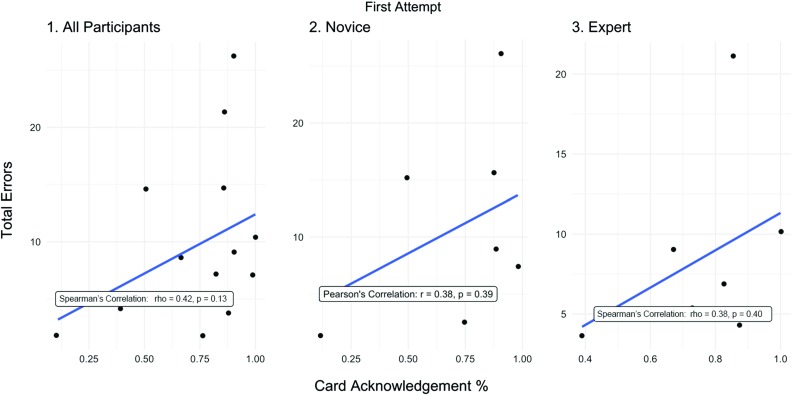
Card acknowledgement % effect on total errors for first attempt. (1) All participants (full dataset), (2) novice only, (3) expert only

**Fig. 3:**
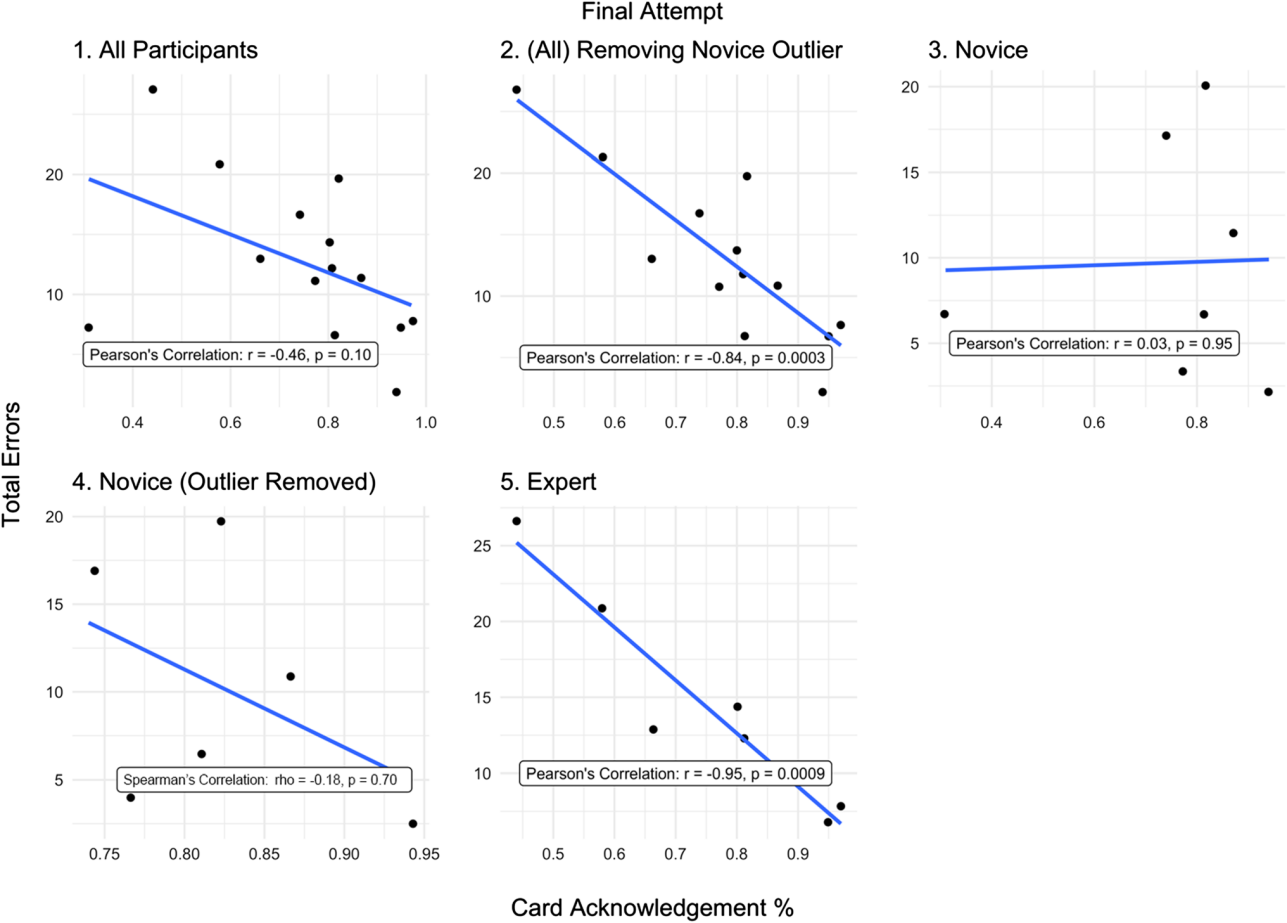
Card acknowledgement % relationship with total errors for the final attempt. (1) All participants (full dataset) included, (2) a clear outlier (a novice) is removed from dataset, (3) novice only, (4) novice only with outlier removed, (5) expert only

Figure 2 shows the correlation between the less demanding stimulus card task (first procedure attempt) and total errors. There is a moderate but statistically insignificant positive correlation between card acknowledgement rate and total errors (*ρ* = 0.42, *p* = 0.13). Similar correlation values exist between novices and experts (novices: *r* = 0.38 [*p* = 0.39] vs experts: *ρ* = 0.38 [*p* = 0.40]). Figure 3 shows the correlation coefficients between card acknowledgement rates and total errors for the final procedure attempt (involving the more demanding card stimulus task). When including all participants, there is a statistically insignificant moderate negative correlation (*r* = − 0.46, *p* = 0.10), however an obvious outlier exists. This outlier is 5.98 SDs (or standard units/deviations) from the mean distance (residual) from the regression line, hence justifying its removal. With this outlier removed, there is a statistically significant strong negative correlation (*r* = − 0.84, *p* = 0.0003). There is a statistically significant strong negative correlation between errors and card acknowledgement rates within the expert group (*r* = − 0.95, *p* = 0.0009). However, no such corresponding correlation exists when only analysing the novice group only (*ρ* = − 0.18, *p* = 0.70).

### Eye tracking metrics

Table 4 presents the group comparison of AOI specific eye tracking metrics: instruments, vital signs, X-ray and stimulus.

**Table 4:** Group comparison: eye tracking metrics on AOIs on the display screens (Bonferroni-corrected alpha values for 15 tests = 0.003 and for four tests = 0.013)

Eye tracking metric	AOI	Novice	Expert	*P*
Dwell % (% of dwelling during performance)	Instruments	4.7 ± 1.6	11.1 ± 4.3	0.006
	Vital signs	1.7 ± 1.8	1.6 ± 2.2	0.65
	X-ray	30.8 ± 17	42.7 ± 8.8	0.13
	Stimulus	4.7 ± 3.9	7.3 ± 3.8	0.24
	Total	41.9 ± 20.4	62.7 ± 10	**0.03**
Fixation % (% of all fixation during performance)	Instruments	3.5 ± 1.4	8.5 ± 3.5	**0.007**
	Vital signs	1.3 ± 1.4	1.4 ± 2.0	0.70
	X-ray	24.8 ± 14.3	34.2 ± 8.7	0.17
	Stimulus	4.0 ± 3.2	6.1 ± 3.2	0.23
	Total	33.5 ± 17	50.2 ± 9.6	**0.04**
First fixation duration (ms)	Instruments	128 ± 45	157 ± 37	0.24
	Vital signs	105 ± 62	157 ± 105	0.31
	X-ray	152 ± 60	200 ± 100	0.24
	Stimulus	124 ± 50	204 ± 99	0.09

Bold text represent *p*-values below the 0.05 threshold

Experts had a significantly larger dwell % (11.1 ± 4.3 vs 4.7 ± 1.6, *p* = 0.006) and fixation % (8.5 ± 3.5 vs 3.5 ± 1.4, *p* = 0.007) on the instruments screen. In addition, experts had a significantly higher totalled dwell % (63 ± 10% vs 42 ± 20%, *p* = 0.03) and fixation % (50.2 ± 9.6 vs 33.5 ± 17, *p* = 0.04).

Table 5 presents the general eye gaze metrics with none being statistically significantly different between the groups.

**Table 5:** Group comparison: general eye tracking metrics

Eye tracking metric	Novice	Expert	*p*
Fixation frequency (fixations/second)	2.4 ± 0.2	2.5 ± 0.3	0.37
Fixation duration (ms)	223 ± 59	251 ± 53	0.36
Fixation dispersion (pixels)	57 ± 18	52 ± 12	1.00
Saccade frequency (saccades/second)	2.9 ± 0.7	2.8 ± 0.5	0.90
Saccade duration (ms)	76 ± 3	78 ± 1	0.40
Saccade amplitude (°)	75 ± 117	21 ± 7	0.30
Saccade latency (ms)	286 ± 67	284 ± 69	0.94

**Fig. 4:**
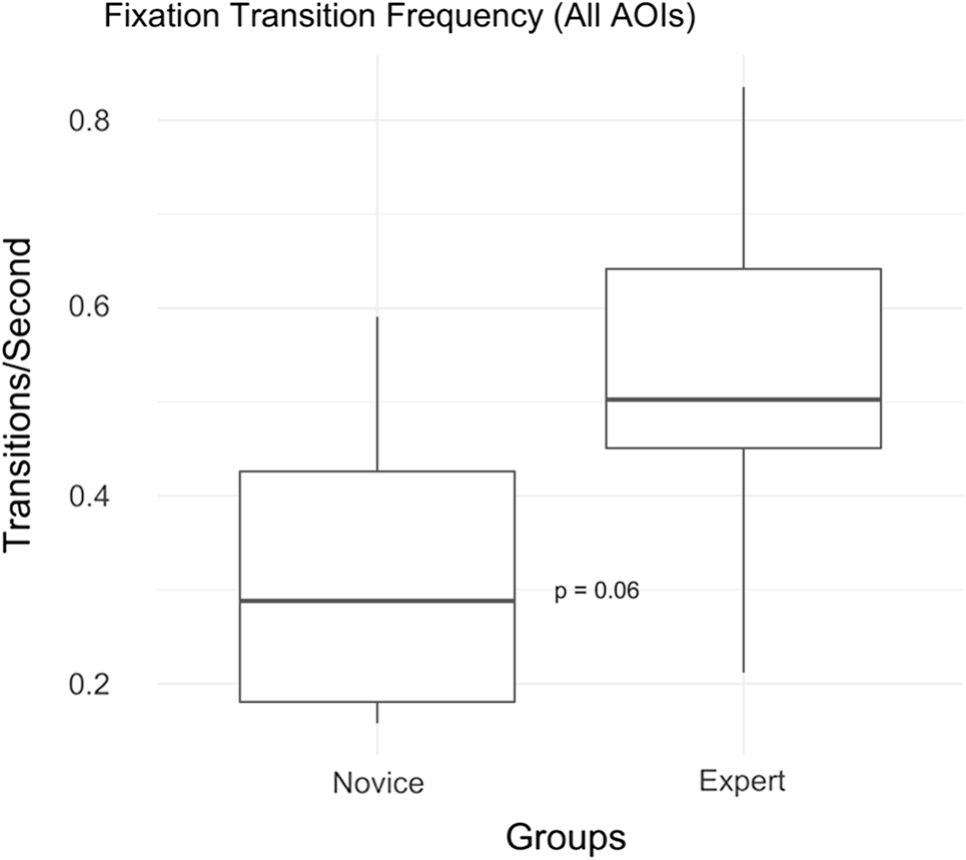
Group comparison for transition frequency over all AOIs

Table 6 shows the fixation transitions between all AOIs. None of the transition count differences are significantly different between groups. Figure 4 shows the group difference for transition frequency: transitions made between any of the AOIs per second.

**Table 6:** Group comparison: fixation transitions between AOIs (display screens)

Fixation start	Fixation end	Novice	Expert	*p*
Instruments	Stimulus	14 ± 20	27 ± 18	0.10
	X-ray	31 ± 30	65 ± 93	0.37
	Vital signs	1 ± 3	2 ± 2	0.52
Stimulus	Instruments	11 ± 15	23 ± 16	0.10
	X-ray	66 ± 73	100 ± 41	0.07
	Vital signs	2 ± 1	4 ± 6	0.60
X-ray	Instruments	35 ± 35	66 ± 96	0.50
	Stimulus	64 ± 75	102 ± 45	0.07
	Vital signs	14 ± 12	15 ± 14	1.00
Vital signs	Instruments	3 ± 4	1 ± 2	0.70
	Stimulus	4 ± 4	3 ± 3	0.60
	X-ray	15 ± 16	15 ± 16	0.80
All	Total transitions	261 ± 244	423 ± 300	0.20
	Transition frequency (transitions/second)	0.32 ± 0.17	0.53 ± 0.20	0.06

### Wristband measurements

**Fig. 5:**
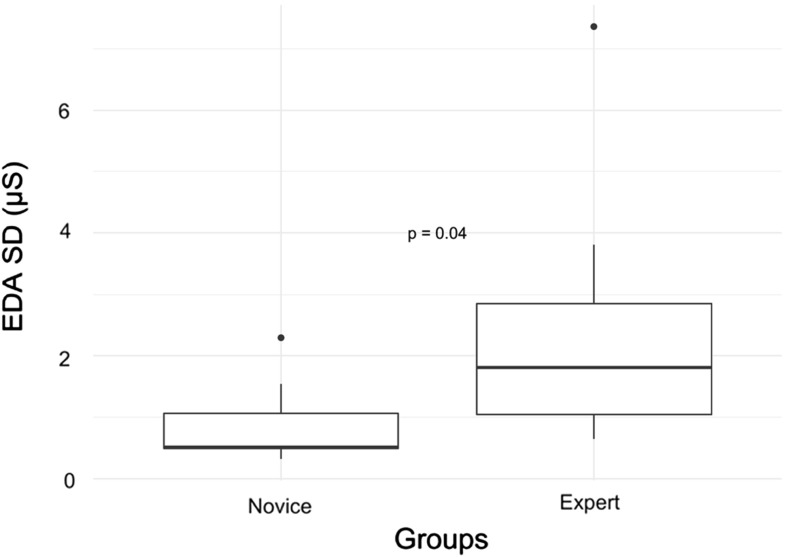
Group comparison of calculated SD for recorded EDA during both attempts

Table 7 presents the statistical analysis of the signals EDA, HRV (i.e. inter-beat intervals), skin temperature and accelerometry (ACC) that are recorded from the wearable E4 wristband. The table provides summary statistics (i.e. mean, min, max and SD) for each signal. No strong statistical correlations were found between the E4 wristband signals and the groups. As shown in Fig. 5, the only insightful significant difference of note is that experts had a larger SD of EDA (2.52 ± 2.38 vs 0.89 ± 0.74 µS, *p* = 0.04). However, if applying Bonferroni-corrected alpha values, then these are not significant findings (Bonferroni-corrected alpha values for 16 tests = 0.003 and for four tests = 0.013)

**Table 7:** Group comparison: psychophysiological measurements from E4 wristband recorded during performances (Bonferroni-corrected alpha values for 16 tests = 0.003 and for four tests = 0.013)

Measurement	Novice	Expert	*p*
EDA			
Mean	1.99 ± 3.16	5.31 ± 4.74	0.13
Min	0 ± 0	0 ± 0	N/A
Max	4.02 ± 2.96	10.85 ± 9.97	**0.03**
SD	0.89 ± 0.74	2.52 ± 2.38	**0.04**
Inter-beat interval (or HRV)			
Mean	0.683 ± 0.148	0.691 ± 0.081	0.80
Min	0.453 ± 0.117	0.464 ± 0.111	0.86
Max	1.096 ± 0.143	1.047 ± 0.109	0.48
SD	0.070 ± 0.016	0.076 ± 0.017	0.50
Skin temperature			
Mean	34.3 ± 1.5	34.1 ± 0.7	0.72
Min	30.3 ± 2.5	26.9 ± 2.3	**0.02**
Max	35.3 ± 2.0	35.6 ± 0.9	0.98
SD	0.6 ± 0.3	1.4 ± 1.0	0.07
ACC			
Mean	63.91 ± 0.05	64.09 ± 0.40	0.28
Min	12.19 ± 2.01	12.68 ± 5.13	0.80
Max	185.08 ± 18.41	174.68 ± 16.94	0.29
SD	5.26 ± 0.26	5.39 ± 1.56	0.84

Bold text represent *p*-values below the 0.05 threshold

## Discussion

This is the first study to use eye tracking and psychophysiological monitoring in this setting. This is also the first study to use a visual stimulus task as a proxy to measure attentional capacity during surgical procedures. This study resulted in several metrics that could be used in a model to automatically discriminate between novices and experts, perhaps leading to assess proficiency in the real setting. Experts had greater dwell time on the X-ray which perhaps indicates their superiority in spatial awareness and coordination; however, this was not statistically significant (*p* = 0.13). Experts also had greater transitions between AOIs which could indicate their intention for more frequent cross-referencing (although this did not quite reach statistical significance, *p* = 0.06). The wristband produced only a small number of metrics that are of interest. Regarding accelerometer-recorded movement, the hands/fingers would be of higher value in future analysis and therefore would necessitate a different type of wearable monitoring tool. Most interestingly, we discovered that card acknowledgement rate during the stimulus task is predictive of the number of handling errors in a procedure (for experts only). It is also interesting to observe the lack of visual attention dedicated to the patient vital signs from both the novice and expert groups (1.6% and 1.7%, respectively).

There is potentially significant value for quantified behaviour during high stakes operations within various environments, from the operating room to the cockpit of a commercial aircraft. Despite the difficult and time-consuming methods required to capture these data, its value when used with machine learning techniques could result in smarter, more responsive environments with intelligent feedback provided to the operators.

Experts complete their first attempt faster than novices; however, in the final attempt, there is little difference. This could be indicative of the confounded effect that the added stimulus task had on the procedural performance—whatever effect it has had on the novice, it could be much more pronounced with experts. Experts have less total errors in their first attempt in comparison with novices, and performance two sees this flipped with the expert committing more errors than the novice. This is a surprising result; however, this result is not statistically significant (*p* = 0.20). One interesting difference is that in the first performance, the expert had 0 ± 0 scraping vessel wall errors reported from the simulator, while in comparison the novice had 1 ± 3. However, when it came to the final attempt, including a much more demanding stimulus task, this inverted despite both groups performing the same case for a second time (in theory, you would expect a better performance), with experts reporting 2 ± 2 compared to novices reporting the same 1 ± 2.

It can be speculated that experts are affected more by the second variation of the stimulus task compared to the novices. Other than this, it can be suggested that either the sample size is too small or that the experts have possibly lost concentration or have demonstrated a waning interest in the challenge by the second attempt.

The stimulus card task produced mixed results when looking at both performances. There were no significant differences in how the groups performed on the additional task while carrying out the procedure. In the second performance, novices improved their correct card acknowledgement rate while the expert % deteriorated slightly changing to a more demanding stimulus task. It could be speculated that the distraction of the cards had a greater impact on experts, perhaps since experts can quickly become ‘in the flow’ given they are more influenced by automatic muscle memory and ‘autopilot’ abilities. Likewise, perhaps the novices are less ‘set’ with the process and additionally, expecting a challenge, therefore able to adapt better. Hence, while experts should have more attentional capacity to undertake an additional task, they are influenced by routine automatic muscle memory which makes it difficult to use an additional task as a proxy for measuring attentional capacity.

The largest effect sizes found when looking for key correlations are that for the final performance two, the expert card acknowledgement % is strongly negatively correlated with the total errors. This relationship for the final attempt is also seen (though not as strongly) with all participants once we have removed one outlier. With the less demanding and less frequent stimulus provided to the participants, card acknowledgement % seems to be weakly positively correlated with total errors. This is consistent in both groups with almost no difference in effect coefficient and *p* value.

This study has suggested that eye tracking could have a role to play in the automated assessment of interventional cardiologist trainees with this type of high-fidelity surgical simulator. The eye tracking metrics have been able to quantify how the expert significantly spends much more visual attention (both with dwell % and its encompassing fixation %) at the display screens compared to the novice. This might be intuitive to those familiar with surgery and may predict it as an expected consequence of superior spatial awareness analogous to an experienced driver (where the expert makes many actions automatically without delay and the need to visually attend to the objects their hands interact with). On average, the expert spends much more of their visual attention looking at the instruments display screen (selecting and changing instruments). We also found that on average the expert will have a higher frequency of fixation transitions between the display screens compared with the novice.

Finally, the attempt to analyse psychophysiological measurements acquired using the E4 wristband has provided little insight. One outcome is that the expert will record a significantly higher SD of EDA for their measurements over time in comparison with the novice. What greater SD in quantified arousal from skin conductance means in a clinical performance setting is up for debate.

### Limitations

Despite the high-fidelity of the laboratory and virtual reality simulator, these data were not recorded in a real clinical environment with real patients. Moreover, we did not fully simulate environmental features such as noise and ongoing staff interactions. We acknowledge that it may never be possible to simulate a procedure that is in par with the real event, since the psychological fidelity is very difficult to emulate. This is a limitation of this study since we are assuming that metrics acquired in simulation settings are transferable to real-life settings. The low sample numbers while understood (feasibility of gathering data from numerous extremely busy operators to partake in a study during a three-month period) hinder what can be inferred from the results. While the sample size is small, each correlation coefficient is accompanied by a statistical test and *p* value that considers the sample size (degrees of freedom) in its calculation. A limitation to this study includes the fact that one of the procedures included a ‘distraction’ of undertaking a secondary unrelated task, i.e. card acknowledgements. In addition, we acknowledge a lack of a proper control group to compare with the procedure that included this additional distracting card acknowledgement task. Also, we must acknowledge that there was no baseline psychophysiological measurement of the participant before the session. For example, context for a participant that was already stressed is not considered or that some participants may have been eager to leave within a certain time, having a rushed effect on their final procedure attempt. Another limitation is that we removed an outlier for a correlation computation because this outlier was 5.98 SDs (or standard deviations/units) from the mean distance (or mean residual) from the regression line; however, often outliers in small samples can be meaningful and removing them can dramatically change results. We acknowledge the limitation of multiple hypothesis tests which increase the likelihood of type 1 errors (false positives) and false discovery rates; however, we have included Bonferroni-corrected alpha values where appropriate. We also acknowledge that participants with prior exposure to the simulation technology can be a confounder in studies that measure performance on a simulator where some subjects have had prior experience of the technology and others have not, which begs the question whether some operator performances are partly influenced by their proficiency with the simulator technology. However, only 7% of subjects had prior experience with the simulator.

### Future work

Some metrics almost statistically discriminate between the two groups but perhaps lack significance due to the low sample numbers. As a result, we have provided guidance in ‘Appendix B’ for future recruitment using power calculations based on the effect sizes in this study. Future studies attempting a similar experimental set-up should consider the length of time provided to participants for practice and familiarising with the surgical simulator. This would reduce the confounder of computer literacy. For further testing of the stimulus card task, other metrics such as mean saccade latency (ms) specific to the stimulus card (from the moment it appears on screen) to the moment it is acknowledged may follow in future work—this would be a more precise measurement of attentional capacity in comparison with the rudimentary count of correctly acknowledging the card. Furthermore, while we only used the procedure errors as detected by the MENTICE VIST simulator, other procedural errors could be classified in future studies, such as those described by Mazomenos et al. [29]. Other future work could determine the extent of which brief prior exposure or proficiency of using the simulator technology can affect the operator’s procedural performance on the simulator?” Put differently, can knowledge of the simulator be a confounder in studies such as the one described in this paper.

Looking beyond the simulation laboratory setting, capturing psychophysiological metrics and measurements in a real clinical environment, while still running a simulation would add to the validity of the data captured. In the case of this procedure, using a simulated operator room with full immersion: leads, scrubs and a theatre team to support the participant. This could drive larger differences between genuine novices and experts. Beyond that, it would seem that this work is linked with a greater goal of creating what could be called ‘smart theatres’.

## Conclusions

This work contributes to the future of sensor-based smart theatres and the ‘quantified physician’ for assessing trainees and operators and to perhaps provide ongoing automated analytical feedback to individuals and teams to drive performance. The study captured a unique dataset with psychophysiological metrics along with a novel measurement of attentional capacity recorded during an important highly skilled clinical procedure. Only a few significant differences between groups have been found when using these metrics: most notably the dwell % and fixation % spent on the display screens. However, the point of this exploratory study is to highlight a number of novel variables that warrant further investigation for assessing proficiency, namely: dwell time on screens, fixation transition frequency between screens, SD of EDA signal and card acknowledgement rates (when using an additional task to measure attentional capacity).

We do acknowledge that this paper mainly focuses on ‘construct validity’ since we wanted to determine whether the metrics can distinguish between novices and experts before providing a more granular analysis which would require a greater number of subjects. Overall, this study provides incentive for further work in the area, with larger sample sizes, a larger range of procedures and using higher fidelity environments.

## Figures and Tables

**Table 8:**
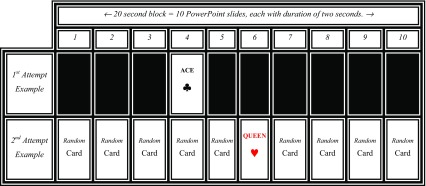
Stimulus task variations: examples of the 20 s blocks with one card to acknowledge

## Appendix A: Card task variation

Table 8 shows the two variations of card task are visualised in their 20 s blocks. These blocks are what maintain the semi-random nature of the card to be acknowledged in the task, while also guaranteeing the participant will have three cards to acknowledge every 60 s.

For this first variation, the playing card does not change to another card in the deck and it was chosen as the ‘Ace of Clubs’. The participant was asked to verbally acknowledge the card with the word ‘clubs’. For the second stimulus task, the participant was provided a constant stream of different playing cards. It was decided that the ‘Queen of Hearts’ would be the card to acknowledge this time. A playing card randomiser was used to select nine random cards to accompany the chosen card in a 20 s block. The participant acknowledged this card with the word ‘heart’.

## Appendix B: Future sample numbers

In advising future studies that investigate a specific metric, we provide the minimum sample numbers per group with the assumption of meeting criteria of statistical significance ($\alpha = 0.05$) and statistical power = 0.9.

### Procedural performance and change of stimulus task

A surprising finding was the increase of total errors by experts in comparison with novices when they repeated the same case but with a much more attentional demanding stimulus card task to attend to (0 ± 8 vs +6 ± 10, *p* = 0.20, power = 0.55). A future study would require 36 subjects per group.

### Eye tracking metrics on AOIs

With metrics on the display screens, some differences were close to statistical significance for the groups and in a future study:

- Dwell % on the X-ray (30.8% ± 17 vs 42.7% ± 8.8, *p* = 0.13, power = 0.68) would require 17 subjects per group.
- Fixation % on the X-ray (24.8% ± 14.3 vs 34.2% ± 8.7, *p* = 0.17, power = 0.68) would require 20 subjects per group.
- First fixation duration on the stimulus (124 ms ± 50 vs 204 ms ± 99, *p* = 0.09, power = 0.69) would require 14 subjects per group.

Other metrics that were close to significance such as total dwell duration and total fixation duration on the instruments screen are strongly correlated with dwell % and fixation % for that AOI and should not be pursued over the %-based metrics. Fixation count on the instruments screen is another but is also strongly correlated with fixation % for the same AOI.

### Fixation transitions between AOIs

None of these metrics were statistically significant between the groups, despite the experts consistently having higher counts. In a future study:

- Instruments » stimulus (14 ± 20 vs 27 ± 18, *p* = 0.10, power = 0.48) would require 24 subjects per group.
- Stimulus » instruments (11 ± 15 vs 23 ± 16, *p* = 0.10, power = 0.77) would require 19 subjects per group.
- Stimulus » X-ray (66 ± 73 vs 100 ± 41, *p* = 0.07, power = 0.56) would require 35 subjects per group.
- X-ray » stimulus (64 ± 75 vs 102 ± 45, *p* = 0.07, power = 0.61) would require 30 subjects per group.
- Total transitions (261 ± 244 vs 423 ± 300, *p* = 0.20, power = 0.59) would require 32 subjects per group.
- Transition frequency (0.32 ± 0.17 vs 0.53 ± 0.20, *p* = 0.06, power = 0.60) would require 13 subjects per group.

### Wristband measurements

The only measurement approaching significance and potentially worth further investigating is SD of skin temperature. It is still debatable what insight this is providing but nevertheless, experts measure a higher skin temperature SD (1.4 °C ± 1.0 vs 0.6 °C ± 0.3, *p* = 0.07, power = 0.56) and a future study would require 17 subjects per group.

### All the above

Using the individual metric recommendations above, if this study was to be repeated without alteration, the minimum number of participants would be 36 subjects per group for a high likelihood of detecting these significant differences (if they truly exist) novices and experts performing simulated coronary angiography.

## Footnotes

1 Vascular Intervention Simulation Trainer: http://www.mentice.com/vist-lab-with-vist-g5.

2 https://www.smivision.com.

3 https://www.empatica.com/e4-wristband.

## References

[1] Kohn L, Corrigan J, Donaldson M (2000). To Err is human: building a safer health system.

[2] Zhang J, Patel VL, Johnson TR (2002). Medical error: is the solution medical or cognitive?. J Am Med Inform Assoc.

[3] Ericsson KA, Krampe RT, Tesch-Römer C (1993). The role of deliberate practice in the acquisition of expert performance. Psychol Rev.

[4] Pedowitz RA, Nicandri GT, Angelo RL, Ryu RKN, Gallagher AG (2015). Objective assessment of knot-tying proficiency with the fundamentals of arthroscopic surgery training program workstation and knot tester. Arthroscopy.

[5] Angelo RL, Ryu RKN, Pedowitz RA, Beach W, Burns J, Dodds J, Field L, Getelman M, Hobgood R, McIntyre L, Gallagher AG (2015). A proficiency-based progression training curriculum coupled with a model simulator results in the acquisition of a superior arthroscopic bankart skill set. Arthroscopy.

[6] Cant RP, Cooper SJ (2014). Simulation in the Internet age: the place of Web-based simulation in nursing education: an integrative review. Nurse Educ Today.

[7] Sliney A, Murphy D (2008) JDoc: a serious game for medical learning. In: Proceedings of the first international conference on advanced computer interaction ACHI 2008, pp 131–136

[8] Persson J, Dalholm EH, Wallergård M, Johansson G (2014). Evaluating interactive computer-based scenarios designed for learning medical technology. Nurse Educ Pract.

[9] Lear R, Riga C, Godfrey AD, Falaschetti E, Cheshire NJ, Van Herzeele I, Norton C, Vincent C, Darzi AW, Bicknell CD, LEAP Study Collaborators (2016). Multicentre observational study of surgical system failures in aortic procedures and their effect on patient outcomes. Br J Surg.

[10] Fong A, Hoffman DJ, Zachary Hettinger A, Fairbanks RJ, Bisantz AM (2016). Identifying visual search patterns in eye gaze data; gaining insights into physician visual workflow. J Am Med Inform Assoc.

[11] Zheng B, Tien G, Atkins SM, Swindells C, Tanin H, Meneghetti A, Qayumi KA, Panton ONM (2011). Surgeon’s vigilance in the operating room. Am J Surg.

[12] Zhou S, Gali R, Paasche-Orlow M, Bickmore TW (2014) Afraid to ask: proactive assistance with healthcare documents using eye tracking. In: Proceedings of the extended abstracts 32nd annual ACM conference on human factors in computing systems—CHI EA’14, pp 1669–1674

[13] Breen CJ, Bond R, Finlay D (2014). An evaluation of eye tracking technology in the assessment of 12 lead electrocardiography interpretation. J Electrocardiol.

[14] O’Meara P, Munro G, Williams B, Cooper S, Bogossian F, Ross L, Sparkes L, Browning M, McClounan M (2015). Developing situation awareness amongst nursing and paramedicine students utilizing eye tracking technology and video debriefing techniques: a proof of concept paper. Int Emerg Nurs.

[15] Just MA, Carpenter PA (1980). A theory of reading: from eye fixations to comprehension. Psychol Rev.

[16] Stiegler MP, Gaba DM (2015). Eye tracking to acquire insight into the cognitive processes of clinicians. Simul Healthc J Soc Simul Healthc.

[17] Asan O, Yang Y (2015). Using eye trackers for usability evaluation of health information technology: a systematic literature review. JMIR Hum Factors.

[18] Suetsugu N, Ohki M, Kaku T (2016). Quantitative analysis of nursing observation employing a portable eye-tracker. Open J Nurs.

[19] Esysenc M, Keane M (1995). Cognitive psychology: a student handbook.

[20] Broadbent D (1981). Selective and control processes. Cognition.

[21] Gallagher A, Satava R, O’Sullivan G (2015). Attentional capacity: an essential aspect of surgeon performance. Ann Surg.

[22] Weaver W (1949). The mathematics of communication. Sci Am.

[23] Smith HV (1992). Is there a magical number 7 ± 2? The role of exposure duration and information content in immediate recall. Irish J Psychol.

[24] Holmqvist K (2011). Eye tracking: a comprehensive guide to methods and measures.

[25] Currie J, Bond RR, McCullagh P, Black P, Finlay DD, Peace A (2018). Eye tracking the visual attention of nurses interpreting simulated vital signs scenarios: mining metrics to discriminate between performance level. IEEE Trans Hum Mach Syst.

[26] McLaughlin L, Bond R, Hughes C, McConnell J, McFadden S (2017). Computing eye gaze metrics for the automatic assessment of radiographer performance during X-ray image interpretation. Int J Med Inform.

[27] Bond RR, Zhu T, Finlay DD, Drew B, Kligfield PD, Guldenring D, Breen C, Gallagher AG, Daly MJ, Clifford GD (2014). Assessing computerized eye tracking technology for gaining insight into expert interpretation of the 12-lead electrocardiogram: an objective quantitative approach. J Electrocardiol.

[28] Bond RR, Finlay DD, Breen C, Boyd K, Nugent CD, Black ND, Macfarlane PW, Guldenring D (2012) Eye tracking in the assessment of electrocardiogram interpretation techniques. In: Computing in cardiology (CinC). IEEE, pp 581–584

[29] Mazomenos EB, Chang P-L, Rippel RA, Rolls A, Hawkes DJ, Bicknell CD (2016). Catheter manipulation analysis for objective performance and technical skills assessment in transcatheter aortic valve implantation. Int J Comput Assist Radiol Surg.

